# Prescribing Patterns and Pharmacotherapeutic Risk Among Community-Dwelling Older Adults in Brazil: A Cross-Sectional Study

**DOI:** 10.7759/cureus.86139

**Published:** 2025-06-16

**Authors:** Isadora F Odebrecht, Ana Beatriz F Nascimento, Diogo N Silveira, Isadora B Fernandes, Caroline L Harada, Gabriel M de Paula, Augusto L Nunes, Rodolfo Barriviera, Paulo R Bignardi, Karen B Fernandes

**Affiliations:** 1 School of Medicine, Pontifical Catholic University of Parana (PUCPR), Londrina, BRA; 2 High School Scientific Research Program, St. James’ International School, Londrina, BRA; 3 Informatics, Federal Institute of Parana (IFPR), Londrina, BRA; 4 Graduate Program of Health Sciences (PPGCS) School of Medicine, Pontifical Catholic University of Parana (PUCPR), Curitiba, BRA

**Keywords:** aging, deprescribing, older adults, pharmacotherapeutics, potentially inappropriate medications

## Abstract

Background

Prescribing for older adults can be particularly challenging, as treatment regimens are often complex in geriatric patients, increasing the likelihood of polypharmacy, escalating the risk of exposure to potentially inappropriate medications (PIMs), and to a pharmacotherapeutic risk. Therefore, this study aims to identify prescribing patterns and the pharmacotherapeutic risk in community-dwelling older adults in Brazil.

Methodology

This cross-sectional study included 504 physically independent older individuals who self-reported sociodemographic characteristics, comorbidities, and data on current pharmacologic therapies. To analyze the pharmacotherapy, all medications used by the patients were recorded, including complementary alternative medications and over-the-counter medications (OTC). The Beers 2023 criteria were used to identify PIMs. Pharmacotherapeutic risk was stratified into two categories based on the presence of PIMs or OTC on the medication regimen.

Results

This study included 504 older adults (mean age = 69.5 ± 6.4 years), 339 women (67.3%), and 165 men (32.7%). Polypharmacy was observed in 136 (26.98%) individuals, while 180 (36.0%) individuals were using PIMs. Moreover, 97 (19.2%) individuals used OTC medication. Notably, 205 (40.7%) older individuals were classified as being at pharmacotherapeutic risk. In logistic regression, individuals with polypharmacy had nearly five times increased risk of being at pharmacotherapeutic risk (odds ratio = 4.9, p = 0.001), after controlling for sociodemographic and clinical variables.

Conclusions

A marked pharmacotherapeutic risk was observed, driven chiefly by polypharmacy among community-dwelling older adults. Thus, medical education regarding rational use of medication and deprescribing practices can help mitigate adverse outcomes in older adults. Moreover, future research should focus on user-centered design of technological solutions to support clinicians and patients in safely managing and discontinuing high-risk medications.

## Introduction

Population aging is one of the greatest public health challenges worldwide. It is estimated that by 2030, one in six people globally will be aged 60 years or older, and this proportion is expected to reach nearly 2.1 billion individuals by 2050 [1]. Prescribing for older adults can be particularly challenging, as treatment regimens are often complex in geriatric patients, increasing the likelihood of polypharmacy, commonly defined as the regular use of at least five medications. This, in turn, increases the risk of exposure to potentially inappropriate medications (PIMs) and the pharmacotherapeutic risk [2-4].

Medical school curriculum often lacks adequate education about PIMs, which contributes to improper prescription. Teaching students and physicians about useful tools, such as the American Geriatrics Society (AGS) Beers Criteria, a widely used list of PIMs that should generally be avoided in geriatrics, is important to improving prescribing practices and promoting safer, patient-centered care [5].

PIMs are medications that should be avoided in older adults because the risk of adverse drug events (ADEs) outweighs the anticipated benefits, particularly when safer and effective alternatives are available [5]. Despite the risks, PIMs continue to be widely used [6]. High rates of PIM use have been observed internationally, with one-third to half of older adults taking one or more PIMs [7,8]. The growing use of PIMs is particularly concerning, as they are associated with poor medication adherence, drug-drug interactions, medication errors, ADEs, hospitalizations, and other complications, as well as higher healthcare costs [9-12].

Previous studies have suggested that disparities in the use of PIMs and over-the-counter (OTC) medications may impact health equality, which is an important indicator of healthcare quality [13]. For these reasons, assessing prescribing patterns and the use of PIMs across different racial, gender, and economic groups is crucial to ensure equitable quality of care and improve patient safety [14,15]. Effective medication management requires advice that is customized to a patient’s unique health circumstances to ensure the delivery of a personalized medication management plan [16].

Considering that pharmacological interventions can be tailored based on the epidemiology of PIMs, this study aims to investigate the prescribing patterns and identify pharmacotherapeutic risks in community-dwelling older adults in Brazil. This can help highlight areas for improvement and ultimately optimize drug therapy and the healthcare system.

## Materials and methods

Study design, ethical aspects, and sample

This cross-sectional study was conducted based on the Strengthening the Reporting of Observational Studies in Epidemiology (STROBE) criteria. This study was approved by the Irmandade da Santa Casa de Londrina Ethics in Research Committee (approval number: 36329214.6.0000.0099). The committee confirmed that the research adhered to the ethical principles outlined in the 1964 Declaration of Helsinki and its subsequent amendments. All participants were required to provide written, voluntary informed consent to confirm their agreement to participate.

This study aimed to evaluate sociodemographic factors and health indicators in older adults. From a population of 43,610 elderly individuals living in the urban area of Londrina, a sample of 378 individuals was set as the minimum needed to represent the target population. The sample consisted of 504 participants recruited from primary care facilities in Londrina, Paraná, Brazil. The sample was randomly stratified based on the proportion of older adults in each region of the city, and participants were evaluated at the university outpatient clinics.

To be included in the study, participants had to meet the following criteria: (1) be any gender; (2) be aged over 60 years; (3) provide voluntary consent and sign the informed consent form; and (4) be physically independent. Exclusion criteria included individuals who had any illness or limitations that prevented them from completing the questionnaires, such as physical and mental disabilities.

Demographic factors, prescribing patterns, and the pharmacotherapeutic risk

Structured questionnaires were used to collect data on sociodemographic characteristics (gender, age, marital status, and educational level), the presence of comorbidities, and the use of chronic medications.

To analyze the pharmacotherapy, individuals were required to bring their medications or their prescriptions. This information was confirmed later, when needed, by either calling the patient to revise their information or double-checking the medical records (when available). Subsequently, the brand names and dosages of all drugs were recorded. Thus, the active substances were identified, and the daily doses of all drugs used were counted. All conventional medicines and complementary alternative medicines (CAMs) were considered. The pharmacotherapy analysis also accounted for whether each medication had been prescribed. After completing the medication inventory, the 2023 Beers Criteria were applied to identify PIMs [5]. Medications were classified as OTC if the patient was taking them without a prescription.

Both the use of PIMs and OTC medications is associated with a potential risk for adverse outcomes. Therefore, if the patient used at least one PIM or OTC medication, the patient was classified as a high pharmacotherapeutic risk, and if the patient was using neither PIM nor OTC medication, the patient was classified as low pharmacotherapeutic risk.

Statistical analysis

SPSS Version 20.0 (IBM Corp., Armonk, NY, USA) was used for statistical analysis. A confidence interval of 95% and a significance level of 5% (p < 0.05) were established for all applied tests. Descriptive analysis was conducted using absolute and relative frequencies of the studied variables. Multivariate analysis (logistic regression) was used to assess potential associations between sociodemographic variables (age, gender, race, education, and economic status), multimorbidity, and polypharmacy with pharmacotherapeutic risk in the study population. A bivariate analysis was first performed to identify candidate variables (p < 0.20) for inclusion in the final model. In the final analysis, only those with a significance level below 5% were retained as relevant.

## Results

This study included 504 older adults, with a mean age of 69.55 (SD = 6.42) years, 339 (67.26%) women, and 165 (32.74%) men. Demographic characteristics of the studied population are shown in Table 1. In our sample, 242 older adults were using at least one medication daily, while 62 (20.39%) individuals were using no medication. Polypharmacy was observed in 136 (26.98%) individuals.

**Table 1:** General characteristics of the study population.

Variables	Absolute frequency (n)	Relative frequency (%)
Gender		
Female	338	67.1
Male	166	32.9
Age range		
60–70 years	299	59.3
>70 years	205	40.7
Education level		
Up to 8 years	458	90.9
>8 years	46	9.1
Economic status		
A + B	90	17.9
C	315	62.5
D + E	99	19.6
Multimorbidity		
No	237	47.1
Yes	267	52.9
Polypharmacy		
No	368	73.1
Yes	136	26.9
Total	504	100

A total of 121 (24.0%) older adults were taking at least one medication deemed inappropriate for cognitive function. Notably, 205 (40.67%) older individuals were classified as being at a high pharmacotherapeutic risk. In multivariate logistic regression, only polypharmacy remained significantly associated with high pharmacotherapeutic risk in the final model (Table 2).

**Table 2:** Bivariate and multivariate analysis (logistic regression) between demographic and clinical variables with high pharmacotherapeutic risk in older adults. OR: odds ratio; CI: confidence interval

Variables	Bivariate analysis	P-value	Multivariate analysis	P-value
	OR (95% CI)		OR (95% CI)	
Age	1.02 (0.99–1.05)	0.09	1.03 (0.99–1.06)	0.07
Sex				
Female	1	0.32	-	-
Male	1.21 (0.83–1.77)		-	-
Race				
White	1	0.98	-	-
Non-White	1.0 (0.83–1.21)		-	-
Education level				
Up to 8 years	1	0.04	1	0.79
>8 years	0.83 (0.70–0.99)		0.76 (0.77–1.22)	
Economic status				
A+ B	1	0.02	1	0.18
C + D + E	1.19 (1.02–1.38)		1.13 (0.94–1.36)	
Multimorbidity				
No	0.94 (0.85–1.04)	0.25	-	-
Yes	1		-	-
Polypharmacy				
No	1	0.001	1	0.001*
Yes	4.92 (3.13–7.71)		4.92 (3.11–7.78)	

Moreover, 180 individuals were using PIMs, representing nearly 36% of the general population. However, some individuals used more than one PIM daily, encompassing 255 inappropriate medications reported. Medications acting on the central nervous system, such as tricyclic antidepressants and benzodiazepines, were reported as the most PIMs used by older adults.

**Figure 1:**
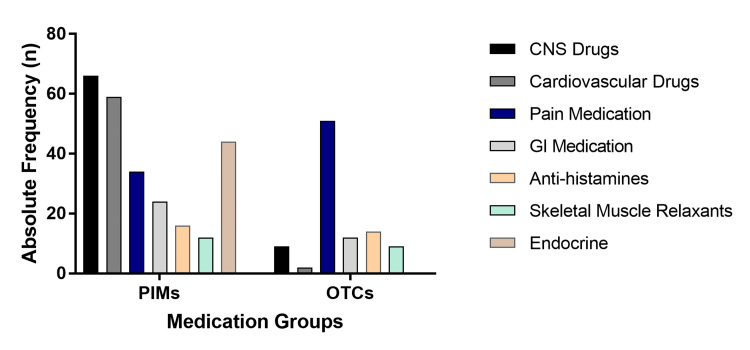
Frequency distribution of the main PIMs and OTC drugs used by the study population. PIM: potentially inappropriate medication; OTC: over-the-counter; CNS: central nervous system; GI: gastrointestinal

Additionally, 97 (19.24%) older individuals used OCT medications. The most common self-prescribed drugs targeted the gastrointestinal system (e.g., proton pump inhibitors) and pain relief (e.g., opioids or nonsteroidal anti-inflammatory drugs). The information regarding the drug types of PIMs and OTC medications mainly used by this study population is shown in Figure 1.

Moreover, 95 (31.25%) individuals reported the use of CAM, most often without medical supervision. The overall pharmacotherapeutic profile is presented in Table 3.

**Table 3:** Frequency distribution of the general pharmacotherapeutic profile of medications used by the study population.

Anatomical therapeutic category (ATC)	Absolute frequency (n)	Relative frequency (%)
Alimentary tract and metabolism	274	18.2
Musculoskeletal system	124	8.3
Nervous system	119	8
Blood	108	7.2
Various	78	5.2
Other ATC classes	143	9.5
Total	1,501	100

## Discussion

Pharmacotherapeutic risk in older adults stems from age-related pharmacokinetic and pharmacodynamic changes, multimorbidity, and the consequent high burden of polypharmacy, all of which increase the susceptibility to ADEs. In this study, polypharmacy alone was associated with nearly a fivefold increase in pharmacotherapeutic risk. Although some studies have reported that PIM increases with age [17], this trend was not observed in our study, perhaps because we included only older adults, making the key contrast one between adults and older adults. Similarly, no associations were found between pharmacotherapeutic risk and sex, educational level, or socioeconomic status, consistent with previous evidence [18]. On the other hand, there is some evidence about the association of low educational level with non-adherence to medication, self-medication, and polypharmacy [17].

The use of polypharmacy and PIMs in older individuals is a significant public health issue. In this study, the polypharmacy rate was nearly 27%, higher than that observed in many other countries, such as Argentina (20.5%) [19], Switzerland (17%) [20], and Singapore (14.5%) [21], suggesting either a greater reliance on pharmacological interventions in our study or more complex patient conditions requiring multiple medications.

Additionally, our study revealed a high prevalence of older adults exposed to PIMs, almost 36% (180 individuals). Some of these patients used more than one inappropriate medication, resulting in a total of 255 PIMs identified among 180 patients. Similarly, Malekzadeh et al. [22] reported that 47.3% of prescriptions in a population-based study in Iran contained at least one PIM according to the Beers Criteria.

Avoiding PIMs is essential to prevent ADEs, thereby reducing pharmacotherapeutic risk in older adults and lowering healthcare costs. It is estimated that 20-30% of all hospital admissions among older adults are related to PIMs, and up to 10% of these cases can be life-threatening or fatal [20].

Another important consideration is that when PIMs are supplied by public health systems, their consumption often increases [23]. Amitriptyline, a tricyclic antidepressant with strong anticholinergic properties, exemplifies this issue. Despite its classification as a PIM, it remains available through many public healthcare programs in developing countries [23,24]. This scenario underscores not only the need for conscious prescribing practices but also the urgency of implementing health policies to decrease PIM use among older adults.

In this study, 97 patients (approximately 20%) reported using OTC medications. The most frequently used classes were analgesics, followed by antihistamines and gastrointestinal agents. Notably, unsupervised OTC use increases the likelihood of PIM exposure, as these drugs can be purchased without medical oversight. All these drug classes are deemed potentially inappropriate according to the Beers Criteria [5] and increase pharmacotherapeutic risk in this population. However, because PIMs can be obtained without medical recommendation, deprescribing is more challenging, as they were never formally prescribed in the first place.

Considering the medication profile, cardiovascular drugs were the predominant category, consistent with previous studies [22]. Notably, antidepressants, anxiolytics, and pain relief medications were also used at high rates. Brazil leads the world in lifetime prevalence of anxiety disorders, ranking fifth globally for depression, which may partially explain this high consumption [25].

Regarding anxiolytics, benzodiazepines were the most used drug class in our study, consistent with previous data [13]. This finding is particularly concerning, given the scarcity of effective pharmacological alternatives for their primary indication, insomnia, and their tendency to induce physical dependence, which makes long-term use and subsequent deprescribing especially challenging.

Deprescribing is an evidence-based intervention to reduce inappropriate polypharmacy and PIMs [26,27]. Deprescribing includes tapering, discontinuing, or withdrawing medications that are either unnecessary or present more risks than benefits to patients [28]. However, deprescribing is not being implemented as often as it should be. Deprescribing is more successful when patients receive clear education about the medication’s adverse effects, such as cognitive impairment, increased fall risk, and dependence. To facilitate this process, clinical practice guidelines and deprescribing algorithms have been developed to help healthcare professionals safely discontinue PIMs [14].

Achieving patient engagement in deprescribing requires a combined caregiver- and patient-centered approach. Maintaining a strong practitioner-patient relationship is crucial for building trust and facilitating effective communication, which is key to shared decision-making and adherence [29]. As reducing PIM use involves multiple factors, interdisciplinary strategies to optimize prescribing, as well as tailored health education and targeted interventions, should be considered to decrease PIMs in older adults [29].

Evidence suggests that increasing physician awareness of PIMs is essential to prevent prescribing cascades, situations in which new medications are prescribed to treat the side effects of other drugs, and the associated pharmacotherapeutic risks [27]. At the health system level, policymakers should ensure that public formularies prioritize safer alternatives for older patients and integrate decision-support tools alongside patient education initiatives to decrease pharmacotherapeutic risk and improve overall care.

Considering the limited availability of tools to support deprescribing [28], future research should focus on both developing and rigorously evaluating the feasibility and user experience of technological solutions in clinical settings, as well as exploring how artificial intelligence and interactive features can enhance their effectiveness in facilitating management of chronic medications use and deprescribing practices.

The cross-sectional design and the lack of comparisons between sociodemographic characteristics and the various classes of potentially inappropriate medications used by this population represent primary limitations of the study; this topic could be explored in future research. One additional limitation could be recall bias, given that medication use was self-reported. Nevertheless, we sought to address this limitation by reviewing pharmacotherapy as needed.

## Conclusions

In our study, 36% of community-dwelling older adults received at least one PIM, most frequently benzodiazepines, antidepressants, and analgesics, and 20% reported unsupervised use of OTC drugs. Such dual exposure heightens the risk of adverse events, drug-drug interactions, and care fragmentation in this vulnerable population. To mitigate these risks, pharmacotherapy reviews and adherence to standardized deprescribing protocols should be implemented across different care settings, followed by targeted patient education on the risks of self-medication. Future research should focus on the design and validation of user-centered digital tools for real-time monitoring, structured tapering, and the safe discontinuation of high-risk medications, thereby promoting safer medication practices among older adults.
